# Web Conferencing Facilitation Within Problem-Based Learning Biomedical Engineering Courses

**DOI:** 10.1007/s43683-020-00020-1

**Published:** 2020-09-02

**Authors:** Sara L. Arena, Yong W. Lee, Scott S. Verbridge, Andre Muelenaer, Pamela J. VandeVord, Christopher B. Arena

**Affiliations:** 1 Department of Biomedical Engineering and Mechanics, Virginia Tech, Blacksburg, VA USA; 2 Department of Pediatrics, Virginia Tech Carilion School of Medicine, Roanoke, VA USA

**Keywords:** Problem based learning, Facilitators, Web-conferencing, Rotating facilitation

## Abstract

Problem-based learning (PBL) has been effectively used within BME education, though there are several challenges in its implementation within courses with larger enrollments. Furthermore, the sudden transition to online learning from the COVID-19 pandemic introduced additional challenges in creating a similar PBL experience in an online environment. Online constrained PBL was implemented through asynchronous modules and synchronous web conferencing with rotating facilitators. Overall, facilitators perceived web conferencing facilitation to be similar to in-person, but noted that students were more easily “hidden” or distracted. Students did not comment on web conferencing facilitation specifically, but indicated the transition to online PBL was smooth. Course instructors identified that a fully synchronous delivery as well as modifications of Group Meeting Minutes assignments as potential modifications for future offerings. Future work will aim to address the perceptions and effectiveness of web conferencing facilitation for PBL courses within an undergraduate BME curriculum, as web conferencing could prove to be another significant breakthrough in addressing challenges of problem-based learning courses.

## Challenge Statement

Problem-based learning (PBL) has been effectively used within BME education^3^,^9^,^13^ and employs a constructivist model of learning where the learner is actively working to construct knowledge.^9^ Typically, students are presented with a complex, open-ended problem and work cooperatively in groups towards a solution.^5^ Instructors, rather than providing traditional lecture-based teaching, serve as facilitators who neutrally probe student knowledge and understanding while also revealing group behaviors by drawing attention to student actions.^2^ Facilitators are critical to PBL as they play an important role in guiding problem-solving actions,^6^,^4^ discourse,^10^ and modeling strategies for learning and thinking.^7^ However, PBL, related to providing facilitation, poses some challenges. Specifically, the greater time investment,^12^ faculty buy-in for this time investment,^12^ and the amount of staffing needed^9^ pose as barriers for implementation of PBL at larger universities with higher enrollments. To address these challenges, a rotating facilitator model of PBL within an introductory BME course was implemented with preliminary findings indicating the model to be beneficial to both students and facilitators.^1^

The rotating facilitator model utilized three to four facilitators per class period, with each facilitator moving between three different groups for direct facilitation. This gave each student group approximately 8–10 facilitation periods per semester while requesting facilitators for only 2 class periods of their time. Prior to the COVID-19 pandemic, the course used in-person instruction, with groups and facilitators within the same physical space. In-person instruction also allowed the full-time course instructors (typically 1–2 faculty and 1–2 graduate teaching assistants) to provide additional facilitation and monitoring of group progress. However, in transitioning to online-only instruction due to the COVID-19 pandemic, instructors were challenged to create a similar PBL experience in an online environment with consideration of possible additional constraints such as accessibility to technology and materials, varied student internet access, and geographically de-localized physical location. As facilitators play a critical role in guiding the learning process,^6^,^4^,^10^,^7^ continuing facilitation was an important consideration in adoption to the online environment. Additionally, due to the number of student groups, direct facilitation by primary instructors would result in a reduced amount of facilitation per group. Therefore, the rotating facilitator model was continued following the online transition through web conferencing (Zoom Video Communications, Inc., San Jose, CA).

## Novel Initiative

This teaching tip focuses on the Spring 2020 offering of an introductory biomedical engineering course that included 86 students across two sections and twenty groups (39 BME majors, 47 BME minor and non-minor students) and 25 facilitators (22 faculty, 3 graduate students). Prior to the COVID-19 pandemic, the course was offered in-person, with 75-min class sessions twice per week. Scheduling of content and deliverables was published on the course website at the beginning of the semester. Additionally, course instructors gave students an overview of the PBL format, including pedagogical advantages of the strategy. Students were also assigned two PBL publications^13^,^8^ for additional insight and understanding of the strategy and facilitator role.

The university announced on March 11th, 2020 that all classes would move online in response to the COVID-19 pandemic after an extended spring break. Following the transition to online delivery and to accommodate that students may be distributed across multiple time zones, the course pivoted to primarily asynchronous instruction, with the exception of facilitation periods. Asynchronous instruction included mini-lectures by guest speakers related to biomedical content (example: inspiration/context for open-ended problem) and by instructors for target skills (example: communication through a technical poster). These mini-lectures were recorded and uploaded for students. A suggested schedule was provided to the students, but students were not required to review lectures on specific days or at specific times. A total of three facilitation periods were offered by synchronous sessions during the pre-pandemic, in-person course time. Group Meeting Minutes were required before and after the transition to online learning whenever students had a facilitation period. A template was provided encouraging groups to include topics discussed, action items, and team member assignment to action items, but no specific format or content was required. These meeting minutes were periodically reviewed by course instructors to monitor groups and their progress. Deliverables were unchanged due to the transition to online except that in-person presentations were modified to be recorded presentations with peer-review by other students.

Following resumption of classes, only two of eighty-six students reported being outside the Eastern Time Zone, and their geographical location did not pose a significant barrier to the scheduled facilitation times. Additionally, six of eighty-six students reported possible issues with internet access, of which use of Wi-Fi hotspots from their mobile device was identified as a potential solution. To our knowledge, only one technical difficulty arose (a facilitator experienced an unstable Wi-Fi connection and resolved the issue with a Wi-Fi hotspot from their mobile device).

Online constrained PBL began with an open-ended problem related to challenges with organ transplantation. The problem positioned students as part of a hypothetical research team tasked with proposing an innovative line of research to be pursued by a non-profit foundation. The two focus areas for students to consider in their proposed work were (1) improving the function of donor and/or compromised organs and (2) tissue engineering approaches for organ structures.

Faculty were recruited to participate as facilitators for at least two class periods. For the open-ended problem after the transition to online, 14 faculty and 2 graduate students from the Department of Biomedical Engineering, College of Veterinary Medicine, and School of Medicine participated as facilitators. A week prior to their assigned facilitation day, facilitators were given a summary guide of *The Tutorial Process*^2^ and the problem and deliverable description.^1^ Additionally, the course instructor or graduate teaching assistant reviewed the main components of facilitation and the rubrics for assessing the teams with facilitators prior to meeting with the student groups.^1^ Facilitators were randomly assigned to groups based upon their reported availability for participation.

Facilitation periods remained on the pre-transition scheduled days. To implement the facilitation, web conferencing through Zoom (Zoom Video Communications, Inc., San Jose, CA) was used. A main session was initiated for all students and facilitators. Students would then be invited to their pre-assigned breakout rooms with their group members (using the pre-assign feature), allowing each group to have their own individual “space” but maintain access to course instructors (by the request assistance feature within the breakout room or by leaving the breakout room to the main room). Facilitators would then be assigned to a specific group breakout room. Nearing the end of the time with this group, a course instructor or graduate teaching assistant would give a time warning to the facilitator that the facilitator would be moving to the next group (each facilitator met with approximately three groups per facilitation period). The course instructor would then re-assign the facilitator to the next group until the end of the facilitation period. Throughout the facilitation period, course instructors and graduate teaching assistants would rotate between groups that were without a facilitator as needed.

## Reflection

### Facilitators

Facilitators were recruited to complete surveys regarding the rotating facilitator model (study reviewed and approved by the Virginia Tech Institutional Review Board). Specifically related to this Teaching Tip, facilitators were asked “Please describe the value of having facilitation in this [web conferencing] format. If possible, please compare to in-person facilitation if applicable”. Of the sixteen facilitators recruited from the second half of the course offering, ten completed the survey. Facilitators generally perceived the web conferencing format as effective as seen from an example comment:

Compared to my facilitator experience in the course last spring, I don't think it really was much different in the web conferencing format… If anything the students were probably a little more laid back and comfortable without having us standing directly in front of them

However, half of respondents also noted they perceived students were able to “hide” or be more distracted in the online format compared to in-person facilitation. An example of this:

The web conferencing appeared to be similar to in-person for my experiences, although it was of course not quite as valuable and easy to communicate since some students didn't have video on and there were difficulty hearing at times. That said, it was overall a positive and a good example of an activity that was well accomplished remotely

Additionally, one facilitator noted that it was more difficult for them in particular to facilitate online:

Besides technology issues (on my end with internet connectivity), it was just a lot harder to engage the students and their conversations were not as robust as the sessions we had in class

### Students

Students were also recruited to complete surveys regarding the rotating facilitator model (study reviewed and approved by the Virginia Tech Institutional Review Board). Students were not directly asked about online facilitation. However, open-response student comments from these surveys as well as from end-of-semester student evaluations of the course (for primary instructor S. Arena) were reviewed for student feedback. Use of end-of-semester evaluations was reviewed by the Virginia Tech Institutional Board and received Not Research Determination.

Of the 86 students recruited for completion of surveys regarding the rotating facilitator model, *n* = 32 responded. Of the *n* = 32 respondents, there were no comments given specifically related to web conferencing facilitation. Future surveys will be modified to address this specifically.

Of the 86 students enrolled, *n* = 77 completed the end of semester student evaluations. Of those, seven respondents provided comments related to the transition to online. These respondents generally agreed that the transition to online went smoothly, as exemplified by the following student comment:

In terms of transition to online due to the COVID-19 Pandemic, this class did a very good [job] of transitioning. Ensuring we had access to zoom and modifying projects so that they could be effectively done remotely made the transition very smooth

Additionally, one student noted:

This class definitely benefits from being in class and not online

### Course Instructors

Course instructors identified two potential modifications to aid in offering PBL online. First, while the selection of predominantly asynchronous delivery was to alleviate potential constraints from the unforeseen and rapid transition to online, a completely synchronous format would be preferred. Synchronous delivery would allow for additional contact points with instructors as well as between groups to engage in conversations about alternative approaches and solutions. Additionally, the asynchronous offering of mini-lectures each included a Discussion Board for students to engage with fellow students, the instructors, and/or guest lecturers on the topic. However, these discussion boards were not utilized. Anecdotally, course instructors thought this was due to the asynchronous option for viewing the lectures and increased effort to engage (as opposed to asking a question in-class or through chat in web conferencing).

Second, not necessarily related to the online format, was modification of Group Meeting Minutes. Course instructors purposefully left Group Meeting Minute requirements open-ended to encourage groups to find a structure that worked for their group. However, this also resulted in a wide range of detail between groups, from half of a page to up to four pages for a given group meeting. Students also tended to use meeting minutes specifically only toward assignment deadlines. As an example, students tended to include items such as “*Assignment* is due in two days” instead of addressing “Team Member 1 will find information on *concept* to include in *assignment*”. More explicit guidance or requirements on level of detail within the Group Meeting Minutes would be beneficial for students in understanding concepts in a broader context as well as beneficial for course instructors in understanding and monitoring of group progress within the online format and facilitation quality. Additionally, in relation to the rotating facilitator model, inclusion of an oral *Executive Summary* assignment by one of the student group members at the start of the facilitation period would be helpful for maintaining flow and increasing engagement within the group meetings.

Web conferencing facilitation, if determined to be similarly effective as in-person facilitation, could be another significant breakthrough in addressing challenges of problem-based learning courses. Specifically within the field of biomedical engineering, the ability to recruit facilitators from outside the department (i.e. medical and health professional programs) as well as physical location (i.e. schools without health-related programs) could drastically expand the pool of potential facilitators. Additionally, because of the interdisciplinary nature and breadth of biomedical engineering, student exposure to a wide range of expertise and fields helps them to obtain a broader perspective as well as understand the importance of a team towards solving biomedical problems.^11^ Future work will be directed to better understand student and facilitator perceptions of in-person versus web conferencing facilitation and potential impact on achievement of learning outcomes.
